# Extracellular Vesicles of *Pseudomonas*: Friends and Foes

**DOI:** 10.3390/antibiotics12040703

**Published:** 2023-04-04

**Authors:** Tania Henriquez, Chiara Falciani

**Affiliations:** Department of Medical Biotechnologies, University of Siena, 53100 Siena, Italy; chiara.falciani@unisi.it

**Keywords:** extracellular vesicles, outer membrane vesicles, *Pseudomonas aeruginosa*, antibiotic resistance, vaccine development

## Abstract

Extracellular vesicles (Evs) are small spherical vesicles capable of transporting molecules (such as proteins, nucleic acids and lipids) from one cell to another. They have been implicated in processes such as cell-to-cell communication, pathogenicity, biofilm formation and metabolism. In parallel, Evs have been proposed as interesting biotechnological tools. In recent years, antibiotic resistance has become a major problem for human health worldwide. A pathogen singled out as among the most lethal antibiotic-resistant organisms is *Pseudomonas aeruginosa*, an important Gram-negative bacterium that has been extensively studied for the production and characterization of Evs. Here, we describe the advances made in the last decade regarding understanding of the role of Evs in the pathogenicity of *Pseudomonas.* We also examine the potential of Evs for the development of new treatment strategies.

## 1. Introduction

Multidrug-resistant pathogens have become a global problem. It was recently estimated that 4.95 million deaths associated with antibiotic resistance occurred in 2019 [1]. Of these, lower respiratory tract infections accounted for more than 1.5 million deaths. According to Murray and colleagues [1], *Pseudomonas aeruginosa* was the sixth leading pathogen responsible for deaths associated with resistance in 2019.

*P. aeruginosa* is a member of the genus *Pseudomonas*, a heterogeneous group containing more than two hundred species of Gram-negative, rod-shaped bacteria capable of colonizing animal and human hosts [2,3]. *P. aeruginosa* is an aerobe that can grow anaerobically using nitrogen as an electron acceptor [2]. It is also an opportunistic pathogen that can live in water, soil, plants and other environments, and grows well between 25 °C to 37 °C, as it is not very sensitive to temperature changes in comparison to other organisms [2]. *P. aeruginosa* can attack specific groups of people, such as those with cystic fibrosis and burns as well as immunocompromised patients, including individuals with cancer, organ transplants, and diabetes, among others [3]. It can also cause other diseases such as endocarditis, pneumonia and urinary tract infections [2], and it is a common cause of nosocomial infections [3]. This ability to colonize a wide range of environments, including the throats, stools and skin of healthy people [2] is due to the large genome of *P. aeruginosa* that codes for a wide set of regulatory elements [4] and a considerable amount of virulence factors, such as pyoverdine (a yellow-green siderophore), pyocyanin (a phenazine pigment), urease, lipases and phospholipase C, exotoxin A, proteases, elastases (LasA and LasB), quorum-sensing molecules and many others [2,3]. Some strains can even produce considerable amounts of an extracellular polyanionic polysaccharide, such as alginate, which is responsible for the mucoid appearance of some colonies, especially those seen in isolates from cystic fibrosis patients [2]. In addition, *P. aeruginosa* contains a wide group of proteins that are implicated in antibiotic resistance, including different types of Resistance-Nodulation-Division, RND, efflux pumps, such as MexAB-OprN, MexXY-OprM and MexEF-OprN [3,5], enzymes inactivating aminoglycosides and β-lactamases [3]. Additionally, in *P. aeruginosa,* some porins, which are proteins involved in the uptake of molecules including antibiotics, have much lower permeability than those of other bacteria [3]. 

In recent years, due to a rise in multidrug-resistant pathogens, there has been growing interest in new strategies for the treatment of infections including phage therapy, bacteriocins, peptide synthesis, and so forth [3]. In this context, extracellular vesicles (Evs) have been identified as important for the colonization of *Pseudomonas* and have attracted attention as interesting tools for vaccine development, antibiotic delivery and the direct killing of pathogens. In this review we explore the role of Evs in the pathogenicity of *Pseudomonas* and evaluate their potential in the development of therapeutics. 

## 2. What Are Bacterial Extracellular Vesicles?

Last century, it was reported that cells from all three domains of life normally secrete vesicles [6]. In the case of bacteria, the first description of extracellular vesicles was by Bishop and Work in 1965 [7,8]. Bacterial extracellular vesicles are spheres that vary in size, from 20 to 400 nm [9,10,11] and are released naturally into the extracellular milieu by bacterial cells. Interestingly, they have been implicated in cell-to-cell communication [9,10,11,12] and other processes due to their ability to transport a wide range of molecules, such as membrane proteins, soluble proteins, lipids and nucleic acids, and deliver them intact to other cells [9,10,11,12].

**Figure 1:**
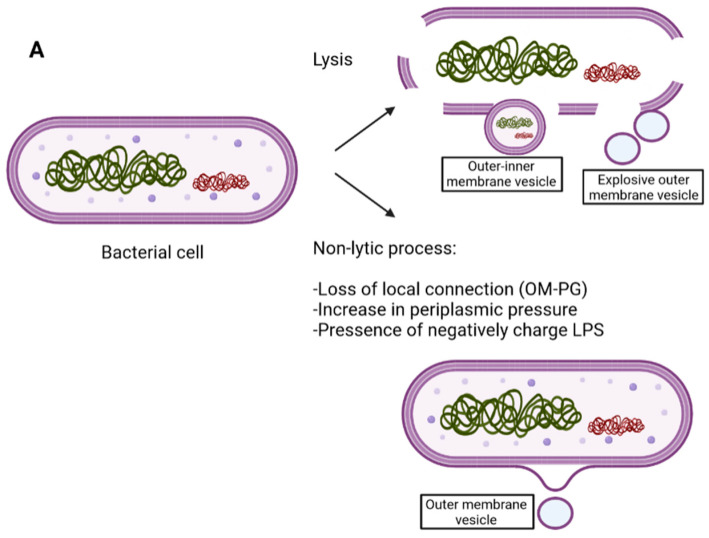

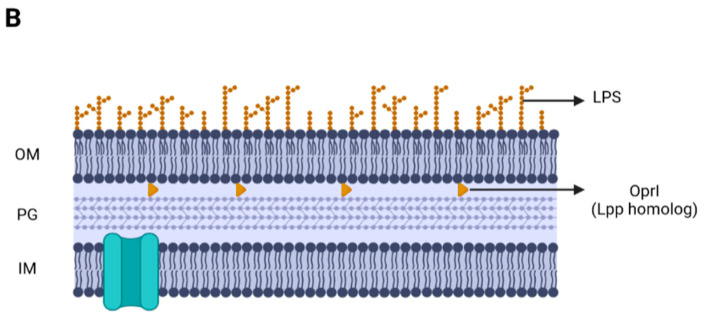
Vesicle formation in *P. aeruginosa*. (**A**) Proposed models for the generation of different types of Evs. The right part of the diagram shows vesicles formed by lysis, OIMV and EOMV in the upper part, and by other mechanisms and OMVs in the lower part. Nucleic acid composition is indicated by green and red intracellular strands. (**B**) Scheme of the components of *Pseudomonas* cells. OM: outer membrane; PG: peptidoglycan; IM: inner membrane; LPS: lipopolysaccharide. Created with https://www.biorender.com/ (accessed on 3 April 2023).

In the case of bacteria, and specifically Gram-negative bacteria, different types of Evs, originally named “blebs” [13], can be described as follows: explosive outer membrane vesicles (EOMVs) resulting from a lytic process, outer membrane vesicles (OMVs), outer-inner membrane vesicles (OIMVs) that contain the outer membrane and the plasma membrane with cytoplasmic content and DNA (Figure 1A), and tube-like membranous structures [10,12,14]. Because this classification was proposed only recently [10], most published research does not distinguish between these different subtypes [12].

## 3. Gram-Negative Structure and EV Biogenesis

The different types of EVs seem to have distinct mechanisms of generation. In the case of OIMVs, some researchers have found evidence of entrapment of DNA and cytoplasmic content in vesicles through protruding inner and outer membranes [15,16]. This seems to fit the model proposed by Kadurugamuwa and Beveridge [17], who suggested that peptidoglycan packing can be weakened by the action of autolysins recovered in the vesicles and that the resulting transient breach in peptidoglycan structure is concomitant with outer and inner membrane blebbing. Ultimately, this phenomenon produces OMVs rich in hydrolytic enzymes with tissue-destructive properties [15]. Other authors describe the formation of OIMVs as a result of endolysin-driven cell lysis that breaks down the peptidoglycan cell wall, causing the shattering of membranes into fragments that self-assemble into OIMVs [10,12,18] (Figure 1A).

Gram-negative bacteria have an inner membrane, a periplasm with layers of peptidoglycan, and an outer membrane, containing lipopolysaccharides (LPS) and proteins, such as membrane-spanning beta-barrel proteins and lipidated proteins [14]. Some outer membrane proteins bind to peptidoglycan non-covalently, whereas others, such as Lpp (a homolog of OprI in *P. aeruginosa*), can form a covalent bond [19,20] (Figure 1B). 

In this context, some of the proposed biogenesis principles for OMVs are: (1) a loss of local connection between peptidoglycan and outer membrane; (2) an increase in negatively charged LPS [12,14]; (3) an increase in periplasmic pressure. We now briefly discuss these mechanisms. For further details on EV biogenesis, other excellent reviews are available [10,12].

(1) Loss of local connection: this mechanism is mainly based on the loss of Lpp or other proteins involved in anchoring the outer membrane and peptidoglycan. In this scenario, the loss of covalent bonds of Lpp to peptidoglycan or changes in protein distribution leading to a lack of protein homogeneity result in the generation of vesicles in the area. This seems to occur because the outer membrane grows more than the peptidoglycan [21].

(2) Negatively charged LPS: LPS comprises three parts: lipid A, the core oligosaccharide, and the O-side chain [22,23]. There are two types of O-side chain, the A-band (also known as common polysaccharide antigen or CPA), which is composed of a polymer of α-D-rhamnose, which is uncharged, and the B-band (or O-specific antigen, OSA), which is composed of repeating units of three to five sugars and is negatively charged [24]. High levels of B-band LPS are thought to be associated with the production of EVs [17,25].

(3) Increase in periplasmic pressure: accumulation of proteins in the periplasmic space (due, for example, to a lack of enzymes involved in their cleavage) has been linked to an increase in turgor pressure which leads to the generation of EVs (Tashiro et al., 2012). An example of this phenomenon is observed when autolysin is removed from *Porphyromonas* [26].

In addition to these proposed mechanisms, other theories have been suggested in other organisms, such as the LPS-sheathed flagellum, that would allow EV formation through their motility [27,28,29]. Altogether, the process leading to EV generation seems to be complex and involves different mechanisms. Because there is no clear pathway, biogenesis may vary depending on species and/or conditions. Further studies are needed to clarify the molecular mechanisms involved in vesicle generation and the sorting of cargo molecules.

## 4. Components of EVs of *Pseudomonas aeruginosa* and Other Species

Extracellular vesicles from *P. aeruginosa* and other *Pseudomonas* species have been studied extensively in recent decades [30]. Table 1 shows the EV components reported for isolates of this species. It should be noted that, although EV composition can be similar to the membranes of the cells producing them, their proportions, as in the case of phospholipids and fatty acids, may be very different [30]. Some studies also suggest that adhesion factors are presumably excluded or at least reduced from OMVs to avoid competition with bacteria for attachment to host cells [13]. 

Over the years, different models have been proposed to explain why nucleic acids may be found in what were originally thought to be vesicles composed of outer membranes because no mechanism yet described accounts for the presence of DNA in the periplasm. A major concern was that the finding of DNA in OMVs could be a research artifact. A possible explanation is that extracellular DNA released from bacteria after lysis is captured by vesicles by a mechanism similar to bacterial transformation [15]. Another model suggested that DNA is integrated into vesicles before release through an unknown mechanism of transport to the periplasm [15], and a third model proposed by Kadurugamuwa and Beveridge concerned “complicated” OMVs [17] (see Section 3 above). Later, this controversy was partly solved by the recognition of different types of vesicles and putative biogenesis principles [10]. In any case, as pointed out by Toyofuku and colleagues, it is difficult to find an explanation other than cell lysis for the presence of fragments of genomic DNA (not plasmid DNA) in vesicles [10]. It is important to mention that DNA can also adhere to vesicles externally. Therefore, if DNA cargo sequencing is required, EVs should first be treated with DNase to remove externally adhered DNA.

In Table 1, it is difficult to compare the results of one study with those of another, as the vesicle components depend on the conditions used to grow the strains [12]. In these reports, vesicles were mostly obtained from strains grown under laboratory conditions. Consequently, even if the strains are clinical isolates, lab-grown strains are likely to have a different EV content from strains reproduced during infection. To clarify EV content during infection, further research into this aspect is needed, especially for important pathogens such as *P. aeruginosa*. Because of these issues, different groups have produced guidelines for isolating and purifying extracellular vesicles [31,32], although most of these recommendations concern eukaryotic cell research. In the coming years, the protocols can be expected to become more uniform.

**Table 1:** Main molecules reported to be carried by EVs from *Pseudomonas aeruginosa*.

Type of Molecule	Molecule Name	Reference
Bacterial protein	OprD, OprE, OprF, OprG, OprH, OprI, PagL, PcoB	[33,34,35]
	Β-lactamases	[36,37]
	FlgK, FlgE, Peptidyl-prolyl cis-trans isomerase, LptD, PilQ, EstA, Gbt, OprC, OpdT, FecA, OpdC, OprB, PslD, LolB, PonA, OprM, LasA, Lipoprotein nlpD/lppB homolog, PilA, OmlA, WspA, among many others.	[34]
	PaAP	[33]
	Cif	[38]
	Cytoplasmic proteins such as: 50S ribosomal proteins, ATP synthase subunit and cytochrome c oxidase *	[34]
Nucleic acid	DNA	[17,39,40]
	RNA, sRNA	[41,42]
Lipid	Glycerophospholipid	[43,44]
	LPS (high levels of B-bands)	[17,25]
Others	Gentamicin (after treatment) *^1^	[17,45]

* Probably outer-inner membrane vesicles. *^1^ Probably explosive outer membrane vesicles or outer-inner membrane vesicles.

## 5. Physiological Roles of Extracellular Vesicles

**Figure 2:**
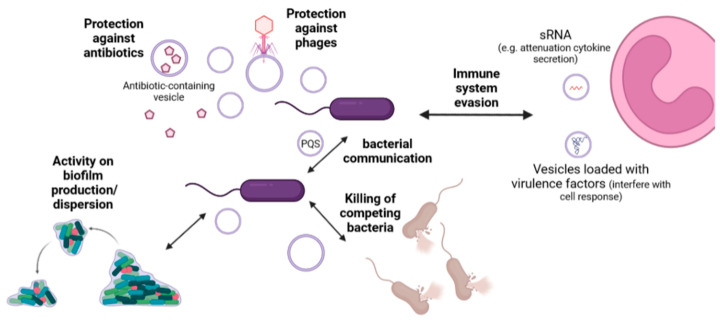
Physiological roles of EVs. Schematic illustration of some of the functions of extracellular vesicles of *Pseudomonas aeruginosa.* Created with https://www.biorender.com/ (accessed on 3 April 2023).

Extracellular vesicles have been implicated in myriad roles in *Pseudomonas* spp. and other organisms. We now explore some of the concepts and findings associated with the pathogenicity of *Pseudomonas* (which are summarized in Figure 2).

Role in cell communication: bacteria synchronize population behaviors using chemical signals known as autoinducers [46]. This process is termed *quorum sensing* and is crucial for pathogenicity. In this context, it has been reported that *P. aeruginosa* EVs carry *Pseudomonas* quinolone signal (PQS), specifically 2-heptyl-3-hydroxy-4(1H)-quinolone, that acts as an autoinducer, mediates iron uptake, chelates ferric iron, and modulates host immune responses [47]. PQS is hydrophobic and is transported inside vesicles which are readily released by the bacterial population [48], thus contributing to the regulation of virulence factors, biofilm formation, iron uptake, cytotoxicity, and outer membrane vesicle biogenesis, among others [49]. The removal of EVs rich in PQS halts the direct communication between cells [47].

The whole mechanism of action and the extent of PQS activity is not completely understood, although it is known to be able to function via the PqsR receptor, a LysR-type transcriptional regulator that can bind to the promoter of *pqsABCDE* operon, and other PqsR-independent pathways [49]. It is also known that PQS affects cells not only by altering the expression of various genes but also by directly binding certain proteins. Interestingly, Lin and colleagues recently showed that PQS in vesicles binds TseF, a type VI secretion system (T6SS) effector protein, and assists in its incorporation into OMVs, facilitating the delivery of iron to cells [50]. Due to the wide-ranging activity of this molecule and its association with vesicles, EV biogenesis could be an interesting target to prevent the colonization of patients by *Pseudomonas* strains. 

In addition to this mechanism, EVs from some pathogens have been reported to fuse with other cells, e.g., EVs from *Pseudomonas* fuse with *Escherichia coli* and *Salmonella* cells [51], and vesicles from enterotoxigenic *E. coli*, a toxin-producing pathogen, fuse with eukaryotic cells [52]. These are examples of bacterial communication and cell communication between kingdoms [53]. 

Role in defense and attack: *P. aeruginosa* is reported to be capable of producing gentamicin-containing vesicles in the presence of sublethal concentrations of gentamicin [17]. This mechanism is suggested to allow protection of the bacterial population as the gentamicin is encapsulated away from its target. A similar phenomenon has been reported in *Pseudomonas putida* IH-2000 and toluene [54,55]. However, whether this is a programmed strategy with physiological significance or just the result of antibiotic action on the cells is unclear. As mentioned in Section 3, compounds like gentamicin destabilize the *P. aeruginosa* membrane, generating blebs, vesicles, and holes in the surface of the cell [56], and are linked to the production of explosive vesicles or OIMVs [12]. Finding the molecular basis that regulates the generation of these vesicles under such conditions could shed some light on the question. In any case, new research indicates that the inhibition of the formation of EVs could contribute to the sensitization of bacteria to antibiotics [57].

Interestingly, it was recently shown that OMVs from *P. aeruginosa* PAO1 can protect the bacterial population against the action of phages such as myovirus KT28 and podovirus LUZ7 [58]. Similar findings have been reported in other organisms [59]. It was suggested that phages can bind to the OMV surface and inject viral genetic material into them, producing an abortive infection [58,59]. This point could be a key consideration when planning in vivo phage therapy strategies.

Besides having signaling properties as already mentioned, PQS contained in vesicles can mediate cytotoxicity against competing microbes. This property has been indicated as an attack strategy [60]. Similarly, EV production in these other strains in response to PQS has been associated with a defense mechanism, as discussed above [30]. One of the first studies on the activity of *Pseudomonas* EVs against other bacterial species was published by Kadurugamuwa and Beveridge in 1996 [45]. Subsequent studies indicated that EVs from these organisms could lyse a broad spectrum of bacteria, including Gram-negative and Gram-positive organisms [61], and that this may be related to the presence of autolysin, which is a 26 kDa murein hydrolase [62] and quinolones [47]. EVs produced in the presence of gentamicin, i.e., gentamicin-containing EVs, are also reported to enhance the lytic effect of normal EVs [45,63]. In this context, EVs are interesting natural antibacterial molecules. However, further research is needed to clarify why EVs kill certain organisms and not others.

Role in biofilm: Biofilms are polymicrobial structures composed of bacteria that form on dry or submerged surfaces [11]. EVs are part of the extracellular matrix of biofilms [64,65]. In some pathogens, EVs are described to promote biofilm formation [66,67,68,69,70]. Studies of *P. aeruginosa* biofilm have shown that EVs are important components [71] and that extracellular DNA is a major part of its cell matrix [72]. Recently, Cooke and colleagues described that OMVs and PQS are generated maximally during *Pseudomonas* biofilm dispersion [73]. The authors also reported that purified OMVs showed lipase, nuclease, and protease activities, which is presumably useful for the extracellular degradation of lipids, proteins, and DNA. This mechanism would facilitate the breakdown of matrix components and cell escape [73]. Further studies into the role of EVs in biofilm formation and maintenance are needed.

Role in interaction with host cells: It was recently reported that EVs from *P. aeruginosa* can control the innate immune response of epithelial cells from human airways through regulatory sRNA [41]. The role of this sRNA, sRNA52320, was also tested in vivo and found to attenuate cytokine secretion and neutrophil infiltration in the lungs of mice [41]. This seems to be the first description of trans-kingdom regulatory activity of an sRNA contained in EVs. Likewise, in a genome-wide DNA methylation study, Kyung Lee and colleagues reported that EVs from *P. aeruginosa* can alter human lung macrophage methylation patterns by producing differential CpG methylation [74] and that some of the CpG affected was associated with cytokines, such as CSF3 [74]. These results indicate that the role of bacterial EVs and their cargo is more complex than previously thought. 

In other pathogens, such as *Helicobacter pylori*, it has been suggested that vesicles loaded with virulence factors in contact with host cells during lifelong infections could allow the bacteria to stay “at a safe distance” from cytotoxicity but still access nutrients and interfere with host immune/cell responses [75]. Based on this, it would be interesting to analyze whether EVs play a similar role in recurrent *Pseudomonas* infections as in chronic lung disease, where the relapse of prior infections seems to be the main cause of disease recurrence [76].

## 6. Extracellular Vesicles as Biotechnological Tools

Extracellular vesicles have been the subject of different therapeutic approaches against infections (Table 2).

Vaccine development: From an immunological point of view, EVs are interesting molecules as they possess pathogen-associated molecular patterns, better known as PAMPs, and can mimic a bacterium without causing the disease [11]. They also have natural adjuvant properties, can strongly induce adaptive immune responses [77,78], and can be easily phagocytized and processed by antigen-presenting cells [11,77]. The most representative and successful vaccines based on EVs are those against *N. meningitidis* group B [11,79]. Studies with EVs from *Pseudomonas* show that LPS and proteins from EVs are recognized and can induce a strong immune response. Banadkoki and colleagues recently found that OMVs from *P. aeruginosa* conjugated with diphtheria toxoid, formulated with alum adjuvant, generated effective protection when used as a vaccine in a mice burn model for *Pseudomonas* infection [80]. Similarly, Zhang and colleagues showed that immunization with EVs from *Pseudomonas,* formulated with aluminum phosphate adjuvant, protected mice challenged with *P. aeruginosa* in a model of acute lung infection, reducing cytokine secretion, bacterial colonization, and tissue damage [81]. Likewise, Ito and colleagues successfully used EVs from *P. aeruginosa* to develop a prophylactic and therapeutic vaccine for corneal infections in C57BL/6 mice [82]. In their study, the authors injected the EVs intramuscularly into mice before infecting the cornea. In parallel, sera derived from ICR (Institute of Cancer Research) mice immunized with *P. aeruginosa* EVs were injected intraperitoneally into naïve C57BL/6 mice before infecting the cornea. It was found that both passive and active immunization significantly affected the infection process by *P. aeruginosa.* Additionally, it was reported that using the immune sera as a topical treatment for the cornea decreased the bacterial load and clinical score [82]. Interestingly, although the use of LPS in vaccines can have considerable side effects, in this study, the administration of EVs without modification or removal of LPS did not seem to generate unwanted effects [82]. Most studies using well-characterized *Pseudomonas* antigens for vaccine development in the last 50 years have been unsuccessful [83]. Only a small number of them have entered clinical trials. However, there is no vaccine licensed for commercial use and there are currently no ongoing clinical trials [83]. In this context, formulations containing EVs seem promising for finally generating a clinically approved vaccine for this pathogen [80].

Vehicles for drug delivery: EVs are excellent vehicles for the simultaneous delivery of hydrophobic and hydrophilic molecules. PQS is an example of a hydrophobic molecule transported by EVs in *P. aeruginosa.* As described by Mashburn and Whiteley, most PQS in this species is released in association with EVs [47]. In other organisms, such as *Paracoccus denitrificans,* the release of hydrophobic molecules (N-hexadecanoyl-L-homoserine lactone) associated with EVs has also been reported [84]. This natural property of EVs holds promise for more efficient delivery of hydrophobic antibiotics, which tend to show a low availability and limited absorption [85].

Nevertheless, due to the immunogenic properties of EVs, it is still difficult to use them efficiently as drug delivery vehicles in vivo. Attempts have been made to reduce the toxicity of LPS: for example, deletion of genes coding for some enzymes involved in the synthesis of Lipid A is reported to result in strains with lower endotoxicity [86,87]. Other points to be improved for successful employment are the low yield after purification, the correct incorporation of specific molecules, and the targeting of specific types of cells [9]. The common EV purification protocol (by ultracentrifugation after bacterial culture) produces a low yield of vesicles which are not optimal for biotechnological applications [9]. In this context, groups have been working on generating modified strains with higher vesiculation, with excellent results [88,89]. Regarding vesicle loading with desired molecules, different approaches have been used. As mentioned above, Kadurugamuwa and Beveridge incubated a bacterial culture in the presence of gentamicin in order to obtain antibiotic-containing vesicles [17]. Other groups have used this method, although active loading techniques, such as electroporation and sonication, have also been used [9]. In the case of cell type-specific targeting, specific proteins or antibodies could be localized on the surface of vesicles to promote their interaction with epitopes present on a desired cell line or bacterium. This technique is not commonly employed, although it could be a promising strategy for the targeted delivery of antibiotics [9]. 

Direct killing of pathogens: As mentioned above, EVs from certain organisms, such as *P. aeruginosa*, are reported to kill competing Gram-positive and Gram-negative bacteria, having virulence factors such as murein hydrolase [61]. In this regard, Cooke and colleagues recently showed that the ability to kill *Staphylococcus* cells varies considerably between *P. aeruginosa* strains and shows a negative correlation with size [90]. Likewise, Berleman and colleagues reported that EVs from *Myxococcus xanthus* killed *E. coli* cells [91], which, in this case, was linked to some putative hydrolytic enzymes that allowed the vesicles to fuse with the target cells [91]. However, there is still no complete molecular characterization of this mechanism, and the range of species involved is unknown. As in the case of the use of extracellular vesicles to deliver drugs, their use for killing pathogens is limited by LPS toxicity, and the low yield of purification protocols, among other issues [9]. Much work is still needed to optimize a strategy for biotechnological applications. 

## 7. Conclusions

The evidence produced in recent decades of research on extracellular vesicles indicates that these molecules are important for many bacterial processes, including bacterial-host interaction, and hold promise for biotechnological applications, especially for strategies against multidrug-resistant pathogens (e.g., as vaccine or drug delivery systems). Understanding their complete physiological role and pathogenic implications is essential for their future deployment. 

The research field of extracellular vesicles is moving fast, and several excellent articles on the topic are produced every year. In the present review, instead of describing all the processes associated with vesicles in *P. aeruginosa* and other species, we delineated what seem to be some of the main roles of EVs in *Pseudomonas* pathogenicity and highlighted some areas with potential for the development of future treatments. For example, investigating how PQS molecules are packed into and released from vesicles could lead to a strategy to block the action of quinolone and undermine part of the quorum-sensing and virulence of the *Pseudomonas* strains. Likewise, identifying the mechanisms responsible for EV generation and how they are regulated could lead to treatments that block the process. In this case, combining the new drugs with antibiotics would probably increase the efficacy of treatment, as no more vesicles containing antibiotics would be released to protect or detoxify the bacteria cells. In this context, exciting new research is being produced. However, as already mentioned, questions remain about the physiological significance and mechanisms involved in vesicle generation, which can only be answered through additional research. 

Here, we also provided information about some of the latest findings and advances concerning *Pseudomonas* EVs and vaccine development. The studies performed in the last ten years seem very encouraging and indicate that vesicles from a set of *P. aeruginosa* strains could eventually have successful clinical applications, which would be particularly important for high-risk groups such as cystic fibrosis patients. The use of vesicles to deliver drugs also seems promising, though, of course, some problems need to be tackled before widespread use. There has been promising new research into the generation of less antigenic LPS. The difficulty of large-scale purification of vesicles with a high yield and homogeneous size and composition remains to be solved. Finally, we discussed using native bacterial vesicles to kill pathogens directly. This strategy is the least developed of all, as the lack of detailed understanding of vesicle generation and the molecular basis of vesicle-cell recognition is delaying its progress. This strategy is also limited by problems regarding the use of vesicles as a drug delivery system. Nevertheless, its future potential as a bactericidal treatment should not be ruled out.

Altogether, the use of extracellular vesicles as biotechnological tools is a promising approach that could lead to new effective therapies against multidrug-resistant pathogens, especially *P. aeruginosa*.

## Figures and Tables

**Table 2:** Summary table of some of the advantages of using EVs as biotechnological tools.

Application	Advantages	Reference
Vaccines	EVs can mimic a bacterium without causing the disease.	[11]
	Have natural adjuvant properties and induce adaptive immune responses.	[77,78]
	EVs can be easily phagocytized and processed by antigen-presenting cells	[11,77]
Vehicles for drug delivery	EVs are strategic vehicles for the simultaneous delivery of hydrophobic and hydrophilic molecules	[9]
Direct killing of pathogens	EVs can kill competing Gram-positive and Gram-negative bacteria, using molecules such as murein hydrolase	[62]
